# 1,2-Dihydr­oxy-2-(3-methyl­but-2-en­yl)-3-oxo-2,3-dihydro-1*H*-indene-1-carboxylic acid monohydrate

**DOI:** 10.1107/S1600536810000516

**Published:** 2010-01-13

**Authors:** Acácio Ivo Franscisco, Gleiciani de Q. Silveira, Jackson A. L. C. Resende, Tatiane L. Balliano, Valéria R. S. Malta, Antonio Ventura Pinto

**Affiliations:** a Instituto de Química, Universidade Federal Fluminense, Centro, CEP 24020-150, Niterói, RJ, Brazil; b Instituto de Química e Biotecnologia, Universidade Federal de Alagoas, CEP 57072-970, Maceió, Al, Brazil; c Núcleo de Pesquisas em Produtos Naturais, Universidade Federal do Rio de Janeiro, Ilha do Fundão, CEP 21944-971, Rio de Janeiro, RJ, Brazil

## Abstract

The title compound, C_15_H_16_O_5_·H_2_O, is an inter­mediate of the Hooker oxidation reaction, used for the synthesis of 2-hydr­oxy-3-(2-methyl­prop-1-en­yl)naphthalene-1,4-dione (nor-lapachol). The packing in the crystal structure is arranged by an O—H⋯O hydrogen-bonded network along the [100] and [010] directions. Each organic mol­ecule is linked to four other mol­ecules *via* the hydr­oxy groups. The water solvent mol­ecule is connected to carboxylic acid groups by three hydrogen bonds.

## Related literature

For a related structure, see Cunningham *et al.* (1999 ▶). For information on the mechanism of the Hooker oxidation reaction, see: Hooker (1936 ▶); Hooker & Steyermark (1936 ▶); Fieser & Fieser, (1948 ▶); Fieser & Bader (1951 ▶); Fieser *et al.* (1936 ▶); Lee *et al.* (1995 ▶). 
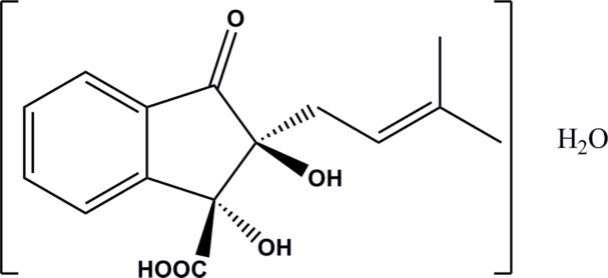


## Experimental

### 

#### Crystal data

C_15_H_16_O_5_·H_2_O
*M* *_r_* = 294.29
Monoclinic, 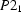

*a* = 9.5514 (7) Å
*b* = 5.7762 (5) Å
*c* = 13.1324 (9) Å
β = 92.126 (12)°
*V* = 724.03 (10) Å^3^
*Z* = 2
Mo *K*α radiation
μ = 0.11 mm^−1^
*T* = 298 K
0.21 × 0.15 × 0.09 mm

#### Data collection

Enraf–Nonius FR590 diffractometer
Absorption correction: multi-scan (*SADABS*; Sheldrick, 1996 ▶) *T*_min_ = 0.981, *T*_max_ = 0.991
18985 measured reflections
1827 independent reflections
1490 reflections with *I* > 2σ(*I*)
*R*_int_ = 0.034

#### Refinement

*R*[*F* ^2^ > 2σ(*F* ^2^)] = 0.037
*wR*(*F* ^2^) = 0.093
*S* = 1.06
1827 reflections
195 parameters
1 restraint
H-atom parameters constrained
Δρ_max_ = 0.15 e Å^−3^
Δρ_min_ = −0.17 e Å^−3^

### 

Data collection: *COLLECT* (Nonius, 2004 ▶); cell refinement: *DIRAX/LSQ* (Duisenberg, 1992 ▶); data reduction: *EVALCCD* (Duisenberg *et al.*, 2003 ▶); program(s) used to solve structure: *SHELXS97* (Sheldrick, 2008 ▶); program(s) used to refine structure: *SHELXL97* (Sheldrick, 2008 ▶); molecular graphics: Mercury (Macrae, 2006 ▶) and *ORTEP-3* (Farrugia, 1997 ▶); software used to prepare material for publication: *SHELXL97*.

## Supplementary Material

Crystal structure: contains datablocks global, I. DOI: 10.1107/S1600536810000516/lh2966sup1.cif

Structure factors: contains datablocks I. DOI: 10.1107/S1600536810000516/lh2966Isup2.hkl

Additional supplementary materials:  crystallographic information; 3D view; checkCIF report

## Figures and Tables

**Table 1:** Hydrogen-bond geometry (Å, °)

*D*—H⋯*A*	*D*—H	H⋯*A*	*D*⋯*A*	*D*—H⋯*A*
O1—H1⋯O2^i^	0.82	1.93	2.729 (2)	166
O2—H2⋯O1^ii^	0.82	2.08	2.846 (2)	155
O4—H4⋯O1*W*^ii^	0.82	1.72	2.520 (3)	164
O1*W*—H1*B*⋯O5^iii^	0.84	1.96	2.785 (3)	167
O1*W*—H1*A*⋯O5	0.84	2.05	2.884 (3)	173

Symmetry codes: (i) 
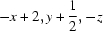
; (ii) 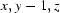; (iii) 
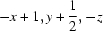
.

**Fig. 1.**
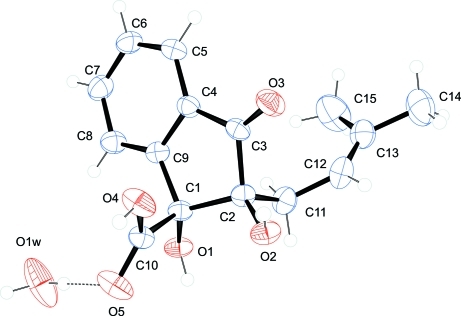
: The molecular structure of (I) with displacement ellipsoids are drawn at the 50% probability level. A hydrogen bond is shown as a dashed line.

**Fig. 2.**
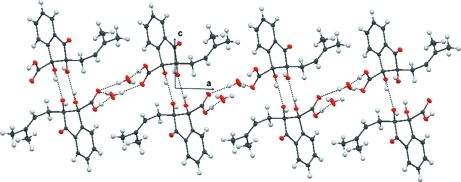
: A packing diagram of (I) (Macrae et al., 2006). Hydrogen bonds are shown as dotted lines.

## supplementary crystallographic information

### Comment

For many years investigations on the Hooker intermediate were object of preparation of nor-lapachol (Fieser *et al.*,  1936), principally due to the different oxidation mechanism (Lee *et al.*,  1995) in which the substrate 2-hydroxy-3-(3-methylbut-2-enyl)naphthalene-1,4-dione) (lapachol) undergoes rearrangement into nor-lapachol (Fieser *et al.*, 1951). This Hooker oxidation reaction is applicable to a large number of hydroxynaphthoquinones with side chains in the quinone ring (Hooker, 1936; Hooker & Steyermark, 1936).

Although spectroscopic data (NMR and elemental analysis) has indicated (Fieser *et al.*, 1948) the likely structure of the intermediate, X-ray diffraction study has never been performed. We report herein the synthesis and the crystal structure of the title compound, (I). An ORTEP-3 (Farrugia, 1997) drawing of (I) is shown in Fig. 1, and selected geometric parameters are presented in Table 1. The five-membered ring adopts an envelope conformation [q2 = 0.260 (2) Å e φ2 = -147.3 (5)°]. The ring is stretched and this is reflected in the larger bond length of C1—C2, like in the oxoindane ester methyl trans-2-(trans-4-tert-butylcyclohexyl)methyl-2,3-dihydroxy- 1-oxoindan-3-carboxylate (Cunningham *et al.*,  1999). The crystal packing is stabilized by five hydrogen bonds (Table 1) forming a hydrogen-bonded network along the [010] and [100] directions (Figure 2).

### Experimental

Lapachol (10.0 g, 41.3 mmol) in THF (70 ml) was added to a solution of Na_2_CO_3_ (4.8 g, 45.3 mmol) in water (100 ml). The mixture was refluxed under N_2_ and when the temperature reached 316K , H_2_O_2_ (32 ml) was added. The reaction mixture remained under reflux for one hour and after this period it was cooled to 283K. Then conc. HCl was added until appearance of a white precipitate, which was filtered under vaccum [yield; 81%, 493-494K, lit. (Fieser *et al.*, 1951): 492-493K].

### Refinement

The hydrogen atoms of the water were placed at calculated positions and other H atoms C—H = 0.93–0.97 Å and O—H (hydroxyl group) = 0.82Å were placed into the calculated idealized positions. All H atoms were refined with fixed individual displacement parameters [*U*_iso_(H) = 1.2U_eq_ (Csp^2^) or 1.5U_eq_ (Csp^3^) and (O—H)] using a riding model. Due to the absence of anomalous dispersion the The Flack parameter was not refined.

### Crystal data

**Table:**

C_15_H_16_O_5_·H_2_O	*F*(000) = 312
*M**_r_* = 294.29	*D*_x_ = 1.357 Mg m^−^^3^
Monoclinic, *P*2_1_	Mo *K*α radiation, λ = 0.71073 Å
Hall symbol: P 2yb	Cell parameters from 18985 reflections
*a* = 9.5514 (7) Å	θ = 3.1–27.5°
*b* = 5.7762 (5) Å	µ = 0.11 mm^−^^1^
*c* = 13.1324 (9) Å	*T* = 298 K
β = 92.126 (12)°	Prism, colourless
*V* = 724.03 (10) Å^3^	0.21 × 0.15 × 0.09 mm
*Z* = 2	

### Data collection

**Table:**

Enraf–Nonius FR590 diffractometer	1490 reflections with *I* > 2σ(*I*)
graphite	*R*_int_ = 0.034
CCD rotation images, thick slices scans	θ_max_ = 27.5°, θ_min_ = 3.1°
Absorption correction: multi-scan (*SADABS*; Sheldrick, 1996)	*h* = −12→12
*T*_min_ = 0.981, *T*_max_ = 0.991	*k* = −7→7
18985 measured reflections	*l* = −17→17
1827 independent reflections	

### Refinement

**Table:**

Refinement on *F*^2^	Primary atom site location: structure-invariant direct methods
Least-squares matrix: full	Secondary atom site location: difference Fourier map
*R*[*F*^2^ > 2σ(*F*^2^)] = 0.037	Hydrogen site location: inferred from neighbouring sites
*wR*(*F*^2^) = 0.093	H-atom parameters constrained
*S* = 1.06	*w* = 1/[σ^2^(*F*_o_^2^) + (0.0546*P*)^2^] where *P* = (*F*_o_^2^ + 2*F*_c_^2^)/3
1827 reflections	(Δ/σ)_max_ = 0.029
195 parameters	Δρ_max_ = 0.14 e Å^−^^3^
1 restraint	Δρ_min_ = −0.17 e Å^−^^3^

### Special details

**Table:**

Geometry. All e.s.d.'s (except the e.s.d. in the dihedral angle between two l.s. planes) are estimated using the full covariance matrix. The cell e.s.d.'s are taken into account individually in the estimation of e.s.d.'s in distances, angles and torsion angles; correlations between e.s.d.'s in cell parameters are only used when they are defined by crystal symmetry. An approximate (isotropic) treatment of cell e.s.d.'s is used for estimating e.s.d.'s involving l.s. planes.
Refinement. Refinement of *F*^2^ against ALL reflections. The weighted *R*-factor *wR* and goodness of fit *S* are based on *F*^2^, conventional *R*-factors *R* are based on *F*, with *F* set to zero for negative *F*^2^. The threshold expression of *F*^2^ > σ(*F*^2^) is used only for calculating *R*-factors(gt) *etc*. and is not relevant to the choice of reflections for refinement. *R*-factors based on *F*^2^ are statistically about twice as large as those based on *F*, and *R*- factors based on ALL data will be even larger.

### Fractional atomic coordinates and isotropic or equivalent isotropic displacement parameters (Å^2^)

**Table:**

	*x*	*y*	*z*	*U*_iso_*/*U*_eq_	
O1W	0.5721 (2)	0.8481 (4)	0.0536 (2)	0.0823 (9)	
H1A	0.6162	0.7243	0.0448	0.123*	
H1B	0.4867	0.8512	0.0372	0.123*	
O1	0.90465 (17)	0.6665 (3)	0.10377 (11)	0.0322 (4)	
H1	0.9157	0.6392	0.0433	0.048*	
O2	1.01902 (18)	0.1204 (3)	0.09344 (12)	0.0334 (4)	
H2	0.9917	−0.0037	0.1157	0.05*	
O3	1.03440 (19)	0.0001 (3)	0.30071 (13)	0.0403 (4)	
O4	0.73158 (19)	0.1340 (3)	0.14502 (13)	0.0407 (4)	
H4	0.6744	0.0621	0.1092	0.061*	
O5	0.7005 (2)	0.4019 (3)	0.02549 (15)	0.0484 (5)	
C1	0.8810 (2)	0.4586 (4)	0.15549 (16)	0.0268 (5)	
C2	1.0096 (2)	0.2926 (4)	0.16956 (16)	0.0281 (5)	
C3	0.9894 (2)	0.1855 (4)	0.27440 (16)	0.0283 (5)	
C4	0.9048 (2)	0.3500 (4)	0.33153 (16)	0.0288 (5)	
C5	0.8816 (2)	0.3586 (5)	0.43499 (17)	0.0375 (6)	
H5	0.9206	0.2491	0.4795	0.045*	
C6	0.7988 (3)	0.5348 (5)	0.46952 (18)	0.0411 (6)	
H6	0.7827	0.5462	0.5387	0.049*	
C7	0.7396 (3)	0.6945 (5)	0.40303 (18)	0.0411 (6)	
H7	0.6842	0.8122	0.4282	0.049*	
C8	0.7608 (2)	0.6834 (5)	0.29942 (17)	0.0349 (5)	
H8	0.7197	0.7907	0.2548	0.042*	
C9	0.8446 (2)	0.5086 (4)	0.26450 (16)	0.0270 (5)	
C10	0.7606 (2)	0.3286 (4)	0.10134 (17)	0.0282 (5)	
C11	1.1452 (2)	0.4353 (5)	0.1714 (2)	0.0373 (6)	
H11A	1.1624	0.4881	0.1029	0.045*	
H11B	1.1333	0.5709	0.2138	0.045*	
C12	1.2692 (3)	0.3023 (5)	0.2109 (2)	0.0428 (6)	
H12	1.2888	0.1649	0.1774	0.051*	
C13	1.3534 (3)	0.3581 (5)	0.2876 (2)	0.0420 (6)	
C14	1.4737 (3)	0.2069 (7)	0.3225 (3)	0.0626 (9)	
H14A	1.4621	0.1623	0.3921	0.094*	
H14B	1.5599	0.2908	0.3172	0.094*	
H14C	1.476	0.0711	0.2805	0.094*	
C15	1.3374 (4)	0.5712 (7)	0.3500 (3)	0.0759 (11)	
H15A	1.3521	0.5339	0.4208	0.114*	
H15B	1.2447	0.6326	0.3387	0.114*	
H15C	1.405	0.6844	0.3307	0.114*	

### Atomic displacement parameters (Å^2^)

**Table:**

	*U*^11^	*U*^22^	*U*^33^	*U*^12^	*U*^13^	*U*^23^
O1W	0.0610 (13)	0.0400 (13)	0.142 (2)	0.0055 (11)	−0.0517 (14)	−0.0227 (15)
O1	0.0497 (9)	0.0228 (9)	0.0242 (7)	−0.0045 (7)	0.0027 (7)	0.0025 (7)
O2	0.0465 (10)	0.0265 (9)	0.0274 (8)	−0.0033 (7)	0.0040 (7)	−0.0043 (7)
O3	0.0463 (10)	0.0360 (10)	0.0382 (10)	0.0086 (8)	−0.0046 (8)	0.0069 (9)
O4	0.0463 (10)	0.0323 (10)	0.0424 (10)	−0.0122 (8)	−0.0154 (7)	0.0085 (9)
O5	0.0588 (11)	0.0386 (11)	0.0458 (11)	−0.0091 (9)	−0.0246 (9)	0.0103 (8)
C1	0.0341 (12)	0.0224 (12)	0.0238 (11)	−0.0019 (9)	0.0006 (9)	0.0017 (9)
C2	0.0334 (11)	0.0246 (11)	0.0263 (11)	−0.0016 (9)	0.0003 (9)	−0.0041 (9)
C3	0.0289 (10)	0.0303 (13)	0.0252 (10)	−0.0023 (10)	−0.0065 (8)	0.0000 (10)
C4	0.0283 (11)	0.0315 (12)	0.0263 (11)	−0.0018 (10)	−0.0027 (9)	0.0004 (10)
C5	0.0386 (12)	0.0475 (16)	0.0259 (11)	0.0041 (12)	−0.0033 (10)	0.0052 (11)
C6	0.0414 (14)	0.0581 (18)	0.0241 (12)	−0.0001 (13)	0.0034 (11)	−0.0045 (12)
C7	0.0426 (13)	0.0433 (15)	0.0380 (13)	0.0036 (12)	0.0089 (11)	−0.0046 (13)
C8	0.0422 (12)	0.0297 (13)	0.0327 (12)	0.0013 (11)	0.0014 (10)	0.0017 (11)
C9	0.0307 (11)	0.0248 (11)	0.0256 (11)	−0.0052 (9)	0.0009 (9)	−0.0005 (9)
C10	0.0349 (11)	0.0226 (12)	0.0269 (11)	0.0015 (9)	−0.0010 (9)	0.0005 (9)
C11	0.0329 (12)	0.0359 (14)	0.0434 (14)	−0.0057 (10)	0.0037 (11)	−0.0034 (11)
C12	0.0330 (12)	0.0399 (15)	0.0558 (16)	−0.0009 (11)	0.0083 (12)	−0.0098 (13)
C13	0.0336 (12)	0.0421 (15)	0.0503 (15)	−0.0053 (12)	0.0028 (11)	0.0000 (13)
C14	0.0361 (14)	0.066 (2)	0.085 (2)	−0.0035 (15)	−0.0045 (14)	0.0077 (19)
C15	0.083 (3)	0.065 (3)	0.078 (3)	0.008 (2)	−0.024 (2)	−0.0182 (19)

### Geometric parameters (Å, °)

**Table:**

O1W—H1A	0.840	C6—C7	1.378 (4)
O1W—H1B	0.840	C6—H6	0.93
O1—C1	1.402 (3)	C7—C8	1.385 (3)
O1—H1	0.82	C7—H7	0.93
O2—C2	1.415 (3)	C8—C9	1.378 (3)
O2—H2	0.82	C8—H8	0.93
O3—C3	1.200 (3)	C11—C12	1.489 (4)
O4—C10	1.297 (3)	C11—H11A	0.97
O4—H4	0.82	C11—H11B	0.97
O5—C10	1.208 (3)	C12—C13	1.306 (4)
C1—C9	1.514 (3)	C12—H12	0.93
C1—C10	1.527 (3)	C13—C15	1.490 (5)
C1—C2	1.564 (3)	C13—C14	1.501 (4)
C2—C3	1.528 (3)	C14—H14A	0.96
C2—C11	1.534 (3)	C14—H14B	0.96
C3—C4	1.471 (3)	C14—H14C	0.96
C4—C9	1.381 (3)	C15—H15A	0.96
C4—C5	1.386 (3)	C15—H15B	0.96
C5—C6	1.376 (4)	C15—H15C	0.96
C5—H5	0.93		
			
H1A—O1W—H1B	118	C9—C8—H8	121
C1—O1—H1	109.5	C7—C8—H8	121
C2—O2—H2	109.5	C8—C9—C4	120.5 (2)
C10—O4—H4	109.5	C8—C9—C1	127.7 (2)
O1—C1—C9	109.97 (18)	C4—C9—C1	111.8 (2)
O1—C1—C10	109.13 (17)	O5—C10—O4	124.5 (2)
C9—C1—C10	109.80 (18)	O5—C10—C1	122.6 (2)
O1—C1—C2	116.27 (18)	O4—C10—C1	112.94 (19)
C9—C1—C2	102.25 (17)	C12—C11—C2	112.8 (2)
C10—C1—C2	109.17 (18)	C12—C11—H11A	109
O2—C2—C3	111.45 (19)	C2—C11—H11A	109
O2—C2—C11	108.25 (18)	C12—C11—H11B	109
C3—C2—C11	109.75 (19)	C2—C11—H11B	109
O2—C2—C1	114.67 (18)	H11A—C11—H11B	107.8
C3—C2—C1	103.27 (18)	C13—C12—C11	126.9 (3)
C11—C2—C1	109.33 (18)	C13—C12—H12	116.5
O3—C3—C4	128.8 (2)	C11—C12—H12	116.5
O3—C3—C2	124.4 (2)	C12—C13—C15	123.7 (3)
C4—C3—C2	106.77 (19)	C12—C13—C14	122.3 (3)
C9—C4—C5	121.5 (2)	C15—C13—C14	113.9 (3)
C9—C4—C3	109.08 (19)	C13—C14—H14A	109.5
C5—C4—C3	129.4 (2)	C13—C14—H14B	109.5
C6—C5—C4	117.7 (2)	H14A—C14—H14B	109.5
C6—C5—H5	121.2	C13—C14—H14C	109.5
C4—C5—H5	121.2	H14A—C14—H14C	109.5
C5—C6—C7	120.9 (2)	H14B—C14—H14C	109.5
C5—C6—H6	119.5	C13—C15—H15A	109.5
C7—C6—H6	119.5	C13—C15—H15B	109.5
C6—C7—C8	121.4 (3)	H15A—C15—H15B	109.5
C6—C7—H7	119.3	C13—C15—H15C	109.5
C8—C7—H7	119.3	H15A—C15—H15C	109.5
C9—C8—C7	118.0 (2)	H15B—C15—H15C	109.5
			
O1—C1—C2—O2	−94.2 (2)	C7—C8—C9—C4	0.1 (3)
C9—C1—C2—O2	146.03 (19)	C7—C8—C9—C1	178.9 (2)
C10—C1—C2—O2	29.8 (2)	C5—C4—C9—C8	1.0 (3)
O1—C1—C2—C3	144.38 (19)	C3—C4—C9—C8	−179.4 (2)
C9—C1—C2—C3	24.6 (2)	C5—C4—C9—C1	−177.9 (2)
C10—C1—C2—C3	−91.7 (2)	C3—C4—C9—C1	1.7 (3)
O1—C1—C2—C11	27.6 (3)	O1—C1—C9—C8	40.0 (3)
C9—C1—C2—C11	−92.2 (2)	C10—C1—C9—C8	−80.0 (3)
C10—C1—C2—C11	151.53 (18)	C2—C1—C9—C8	164.2 (2)
O2—C2—C3—O3	30.5 (3)	O1—C1—C9—C4	−141.13 (19)
C11—C2—C3—O3	−89.4 (3)	C10—C1—C9—C4	98.8 (2)
C1—C2—C3—O3	154.2 (2)	C2—C1—C9—C4	−17.0 (2)
O2—C2—C3—C4	−148.40 (18)	O1—C1—C10—O5	0.7 (3)
C11—C2—C3—C4	91.7 (2)	C9—C1—C10—O5	121.3 (2)
C1—C2—C3—C4	−24.8 (2)	C2—C1—C10—O5	−127.3 (2)
O3—C3—C4—C9	−163.8 (2)	O1—C1—C10—O4	−179.68 (19)
C2—C3—C4—C9	15.1 (2)	C9—C1—C10—O4	−59.1 (3)
O3—C3—C4—C5	15.8 (4)	C2—C1—C10—O4	52.3 (2)
C2—C3—C4—C5	−165.4 (2)	O2—C2—C11—C12	−69.4 (3)
C9—C4—C5—C6	−1.6 (4)	C3—C2—C11—C12	52.5 (3)
C3—C4—C5—C6	179.0 (2)	C1—C2—C11—C12	165.1 (2)
C4—C5—C6—C7	1.0 (4)	C2—C11—C12—C13	−123.5 (3)
C5—C6—C7—C8	0.1 (4)	C11—C12—C13—C15	0.3 (5)
C6—C7—C8—C9	−0.7 (4)	C11—C12—C13—C14	178.4 (3)

### Hydrogen-bond geometry (Å, °)

**Table:**

*D*—H···*A*	*D*—H	H···*A*	*D*···*A*	*D*—H···*A*
O1—H1···O2^i^	0.82	1.93	2.729 (2)	166
O2—H2···O1^ii^	0.82	2.08	2.846 (2)	155
O4—H4···O1W^ii^	0.82	1.72	2.520 (3)	164
O1W—H1B···O5^iii^	0.84	1.96	2.785 (3)	167
O1W—H1A···O5	0.84	2.05	2.884 (3)	173

Symmetry codes: (i) −*x*+2, *y*+1/2, −*z*; (ii) *x*, *y*−1, *z*; (iii) −*x*+1, *y*+1/2, −*z*.
